# Effects of Biochar Combined with Nitrogen Fertilizer Application on Pepper Yield, Quality and Rhizosphere Soil Microbial Community Diversity

**DOI:** 10.3390/plants14193082

**Published:** 2025-10-06

**Authors:** Chunyan Wu, Qiyuan Sun, Wei Wang

**Affiliations:** College of Horticulture, Jilin Agricultural University, Changchun 130118, China; wuchunyan@jlau.edu.cn (C.W.); sunqiyuan0825@163.com (Q.S.)

**Keywords:** biochar, nitrogen, fruit quality, soil microbiome

## Abstract

In agricultural systems, excessive application of nitrogen fertilizer often leads to low nitrogen use efficiency and environmental pollution. In order to solve this problem, we studied the synergistic effect of biochar and nitrogen fertilizer on pepper yield, quality and rhizosphere soil health. This study was conducted under a temperate continental monsoon climate in Changchun, China. Using ‘Jinfu 803’ pepper (*Capsicum annuum* L.) as the test material, biochar was prepared from corn straw under oxygen-limited conditions at 500 °C. the comprehensive effects of the combined application of biochar (0, 0.7% soil mass ratio) and nitrogen fertilizer (0, 75, 375, 675 kg/hm^2^ pure nitrogen) on pepper yield, fruit quality, rhizosphere soil physicochemical properties, and microbial community structure were studied. Redundancy analysis (RDA), high-throughput sequencing, and multivariate statistical methods were used to analyze the association patterns between soil environmental factors and microbial functional groups. The results showed that the combined application of biochar and nitrogen fertilizer significantly improved soil porosity (increased by 12.3–28.6%) and nutrient content, increased yield, and improved quality, among which the treatment of 0.7% biochar combined with 375 kg/hm^2^ nitrogen fertilizer (B1N2) had the best effect. Under this treatment, the pepper yield reached 24,854.1 kg/hm^2^, which was 42.35% higher than that of the control (B0N0). Notably, the nitrogen partial factor productivity (PFPN) of the B1N2 treatment (66.3 kg/kg) was significantly higher than that of the corresponding treatment without biochar and was not significantly lower than that of the high-nitrogen B1N3 treatment. The contents of soluble sugar and vitamin C in fruits increased by 51.18% and 39.16%, respectively. Redundancy analysis (RDA) revealed that the bacterial community structure was primarily shaped by soil pH, organic matter, and porosity, while the fungal community was predominantly influenced by alkaline hydrolyzable nitrogen and total nitrogen. Furthermore, the B1N2 treatment specifically enriched key functional microbial taxa, such as Chloroflexi (involved in carbon cycling) and Mortierellomycota (phosphate-solubilizing), which showed significant positive correlations with improved soil properties. In conclusion, B1N2 is the optimal treatment combination as it improves soil physical conditions, increases nutrient content, optimizes microbial community structure, and enhances pepper yield and quality.

## 1. Introduction

Pepper (*Capsicum annuum* L.), belonging to the Capsicum genus of the Solanaceae family, is an annual or perennial crop widely cultivated worldwide [1]. Nitrogen is a key nutrient for crop growth, but its inefficient use in agriculture often leads to environmental pollution and increased production costs. Pepper is an economically important vegetable crop worldwide, has high nutrient demands and is particularly sensitive to nitrogen management practices. As one of the main seasoning vegetables in the residents’ dietary structure, pepper is favored for its unique flavor and rich nutritional value (such as high contents of vitamin C, provitamin A, carotenoids, phenolic acids, and flavonoids) [2,3]. In recent years, the planting area of pepper has steadily increased; in 2019, the global total output of fresh and processed peppers was approximately 42.283 million tons [4]; in 2020, the planting area of pepper in China exceeded 2.1 million hectares, and relying on facility agricultural technologies, annualized production and balanced market supply have been basically achieved [5].

Pepper is a crop with a short life cycle and high yields. Its growth has significant demands for all nutrient elements, such as nitrogen, phosphorus, and potassium, and its dependence on fertilizers is higher than that of many other crops [6,7]. Nitrogen is an essential element for plant growth and development and is crucial for protein synthesis and the structure and function of chloroplasts [8]. However, the widespread application of nitrogen fertilizer is accompanied by potential environmental pollution risks, and improving nitrogen fertilizer use efficiency has become a key issue for environmental protection and sustainable agricultural development [9]. In the cultivation of vegetable crops, excessive application of nitrogen fertilizer often leads to excessive vegetative growth, inhibits root or fruit development, causes fertilizer waste, and causes environmental pollution [10,11], whereas nitrogen deficiency is characterized by yellowing of the lower leaves of the plant and smaller new leaves [12]. Urea is the most commonly used nitrogen source in pepper production. It is rapidly converted into ammonia through ammonification in soil, but the large amount of mineral nitrogen pool produced easily leads to nitrogen loss, and the plant absorption rate is often less than half of the applied fertilizer [13,14,15]. Studies have shown that the combined application of biochar and nitrogen fertilizer can reduce soil nitrogen loss and increase nitrogen retention [16].

Biochar is a carbon-rich byproduct produced by the high-temperature pyrolysis of biomass under oxygen-deficient conditions [17]. In recent years, the application of biochar in agriculture has attracted increasing attention owing to the increasing demand for food and environmental safety. It decomposes slowly, can effectively adsorb and degrade harmful pollutants in soil, and acts as an efficient soil amendment, playing a positive role by improving soil biological, physical and chemical properties (such as enhancing water retention, increasing pH value, increasing porosity, reducing bulk density, etc.) [18,19]. Biochar is considered a promising soil amendment due to its rich microporous structure, high specific surface area, and ability to improve soil fertility and microbial activity. Compared to traditional organic amendments like compost, biochar exhibits greater stability in soil, leading to longer-lasting effects on carbon sequestration and soil structure, though its initial cost can be higher in regions without established production chains. It also contains a large number of macro- and micronutrients necessary for plant growth, which can not only improve soil nutrient levels but also enhance soil organic matter content and microbial activity [20,21]. Given that biochar has a high specific surface area and porosity, its addition has a significant impact on root growth and morphology [22]. In addition, the hydroxyl and carboxyl groups on its surface can protect nutrients through adhesion and cohesion and supply them to the roots in a slow-release manner, thereby promoting plant growth [23,24]. However, the addition of biochar may also decrease enzymatic activity by reducing soil organic matter, thereby inhibiting crop growth [25].

Multiple studies have confirmed that the combined application of biochar and nitrogen fertilizer can significantly increase the yield and fruit quality of crops such as pod pepper [26] and maize [27], and promote the growth of crops such as rapeseed [28] and rice [29]. The synergistic application of biochar and nitrogen fertilizer has become a promising strategy for simultaneously improving crop productivity and soil health. Biochar can slow down nitrogen loss through adsorption, reduce leaching, and provide a favorable habitat for nitrifying and denitrifying microorganisms, thereby improving nitrogen utilization. However, its comprehensive effects on pepper yield, quality, rhizosphere soil physicochemical properties, and microbial community structure remain unclear. In this study, by setting up different proportion schemes of biochar and nitrogen fertilizer combined application, combined with high-throughput sequencing technology, the regulatory mechanism of their effects on the physical and chemical properties of pepper rhizosphere soil and growth and development was systematically analyzed, and the optimal combined application ratio was screened. This study aimed to provide theoretical and technical support for the high-quality and efficient cultivation of pepper.

## 2. Materials and Methods

### 2.1. Materials

The tested pepper variety was ‘Jin Fu 803’, produced by Tianjin Chaoyan Seedling Technology Co., Ltd. (Tianjin, China). Biochar: made from corn stover, provided by Biochar Future Eco-environmental Technology Co., Ltd. (Guangzhou, China); The phosphate fertilizer was (NH_4_)_2_HPO_4_ and the potassium fertilizer was K_2_SO_4_. The experiment was carried out in the greenhouse of the teaching experimental base of Horticulture College of Jilin Agricultural University, and the bulk density of the original soil was 1.14 g/cm^3^, the porosity was 52.27%, the pH was 6.51, the organic matter content was 42.67 g/kg, the alkali-hydrolyzable nitrogen content was 105.44 mg/kg, the available phosphorus content was 106.38 mg/kg, and the Fast-acting potassium content was 45.12 mg/kg.

Physicochemical properties of biochar: the pH was 9.30, the carbon concentration was 45.31%, the phosphorus concentration was 0.243%, the nitrogen concentration was 1.35%, the potassium concentration was 1.18%, and the cation exchange capacity was 24.6 cmol/kg. The tested nitrogen fertilizer was urea (containing N ≥ 46.4%), produced by Ordos New Energy Chemical Co., Ltd. (Ordos, China). The pH of biochar was determined in a 1:20 (*w*/*v*) biochar-water suspension. Carbon, nitrogen, phosphorus, and potassium concentrations were determined using an elemental analyzer, spectrophotometry, and flame photometry, respectively. Cation exchange capacity (CEC) was measured by the ammonium acetate method [30].

### 2.2. Methods

The experiment was conducted at the teaching base of the College of Horticulture, Jilin Agricultural University, from May to September 2024. The greenhouse temperature during the day is 28–35 °C, with a nighttime temperature of 15–18 °C, and the relative humidity is 60–73%. Four nitrogen levels were set (Table 1), which were 0 kg/667 m^2^ of pure nitrogen (N0), 75 kg/hm^2^ (N1), 375 kg/hm^2^ (N2), and 675 kg/hm^2^ (N3), and two biochar levels, which were 0 (B0) and 0.7% (B1) of soil mass, with a total of eight treatments, each treatment was replicated three times, using a randomized block design.

Based on preliminary experiments and previous studies [31], 0.7% biochar application rate was selected, indicating that the amount effectively improved soil properties and would not have a negative impact on crop growth. Four nitrogen levels (0, 75, 375, and 675 kg/hm^2^) were chosen to represent a gradient from deficiency to excess, encompassing the range of common local farming practices and allowing for the identification of optimal and supra-optimal application rates.

Nitrogen fertilizer was applied according to the treatment levels, the application rate of phosphorus fertilizer was 107.85 kg/hm^2^ of P_2_O, and the application rate of potassium fertilizer was 155.1 kg/hm^2^ of K_2_O. The base fertilizer before planting was 30% nitrogen fertilizer, 80% phosphorus fertilizer, and 50% potassium fertilizer, and topdressing was carried out in the late stage. The remaining 70% of the nitrogen fertilizer, 20% of the phosphorus fertilizer, and 50% of the potassium fertilizer were applied as topdressing, which was carried out four times. Seedlings were sown on 2 May, transplanted on 30 June with a plant spacing of 35 cm × 60 cm, and harvested on 10 September. Yield determination was performed starting from the harvest period of the pepper. Each plant was weighed and measured at harvest, and the total yield was recorded. From the beginning of the appropriate harvest period, fruit quality management was determined by daily sampling, which was the same as the local conventional production management.

#### 2.2.1. Determination Indicators and Methods

Yield determination was carried out starting from the harvest period of pepper. Each plant was weighed separately during harvest, and the total yield was recorded.

Nitrogen Partial Factor Productivity (PFPN): PFPN was calculated as the ratio of fruit yield (kg/ha) to the amount of nitrogen fertilizer applied (kg/ha), using the formula: PFPN = Yield/N applied. This metric reflects the overall efficiency of nitrogen fertilizer in producing economic yield.

Soluble Sugar: Soluble sugar content was determined using the anthrone-sulfuric acid method. Briefly, 0.5 g of fresh pepper fruit tissue was homogenized in 10 mL of distilled water and extracted in a boiling water bath for 30 min. The extract was centrifuged, and the supernatant was collected and diluted. An aliquot of the supernatant was reacted with freshly prepared anthrone-sulfuric acid reagent, and the mixture was heated in a boiling water bath for 10 min. After cooling, the absorbance was measured at 620 nm using a spectrophotometer. Glucose was used as the standard for calibration, and the results were expressed as percentage (%) of fresh weight [32].

Soluble protein content was determined using the Coomassie Brilliant Blue G-250 (Shanghai Lanji Technology Development Co., Ltd., Shanghai, China) dye-binding method. Fresh fruit tissue (1.0 g) was homogenized in 10 mL of phosphate buffer (50 mM, pH 7.0). The homogenate was centrifuged at 10,000× *g* for 20 min at 4 °C. An aliquot of the supernatant was mixed with Coomassie Brilliant Blue G-250 (Shanghai Lanji Technology Development Co., Ltd., Shanghai, China) reagent and incubated at room temperature for 10 min. The absorbance was measured at 595 nm. Bovine serum albumin (BSA) was used as the standard, and the results were expressed as milligrams per 100 g of fresh weight (mg/100 g FW) [32].

Vitamin C: Vitamin C (L-ascorbic acid) content was determined by the molybdenum blue colorimetric method. Approximately 2.0 g of fresh sample was homogenized in 10 mL of oxalic acid-EDTA solution (0.1%). The homogenate was centrifuged, and the supernatant was collected. The supernatant was reacted with ammonium molybdate solution and incubated at room temperature for 15 min to form the molybdenum blue complex. The absorbance was measured at 760 nm. A standard curve was prepared using L-ascorbic acid, and the results were expressed as milligrams per 100 g of fresh weight (mg/100 g FW) [32].

Organic Acid: Titratable acidity, as a measure of organic acid content, was determined by the alkali titration method. A 10 g sample of fresh fruit tissue was homogenized in 50 mL of distilled water. The homogenate was filtered, and 10 mL of the filtrate was titrated with 0.1 M sodium hydroxide (NaOH) to an endpoint of pH 8.2 using a digital pH meter. The results were calculated as the percentage of citric acid equivalent (%) [32].

Free Amino Acids: Free amino acid content was determined using the ninhydrin color development method. Fresh sample (2.0 g) was extracted with 10% acetic acid. The extract was centrifuged and filtered. The filtrate was reacted with ninhydrin reagent in a boiling water bath for 15 min. After cooling, the solution was diluted with ethanol, and the absorbance was measured at 570 nm. Leucine was used as the standard, and the results were expressed as micrograms per 100 g of fresh weight (μg/100 g FW) [32].

#### 2.2.2. Soil Sample Collection

Soil samples were collected from the pepper plants on 10 August 2024, at the maturity stage. Rhizosphere soil was collected using the root-shaking method, and random sampling was conducted using the ‘S’-shaped distribution method. The samples were placed in sealed bags, transported back to the laboratory in an ice box, sieved through a 2 mm sieve, and impurities were removed. Each sample was a mixed sample of rhizosphere soil from three pepper plants. Each sample was divided into two parts: one part was used as the soil sample for physical and chemical property analysis, which was air-dried naturally at room temperature, and the other part was stored in a −80 °C refrigerator for DNA extraction and high-throughput sequencing.

#### 2.2.3. Determination of Soil Physicochemical Properties

Soil physicochemical determination was carried out with reference to Bao [30].

The soil pH was determined using the potentiometric method with a soil-water ratio of 1:5, mixed, and allowed to stand.

Porosity: determined using the ring knife method.

Total nitrogen: determined using the Kjeldahl nitrogen determination method.

Alkaline hydrolyzable nitrogen: determined by alkaline hydrolysis diffusion method.

Fast-acting potassium: determined using the ammonium acetate extraction-flame photometer method.

Available phosphorus: determined by the sodium bicarbonate extraction–spectrophotometer colorimetric method.

Organic matter: determined using the potassium dichromate volumetric method.

The cation exchange capacity (CEC) was determined using the sodium acetate method.

Exchangeable sodium (ENa^+^) was determined using NH_4_OAc-NH_4_OH flame photometry.

The soil exchangeable sodium percentage (ESP) was calculated using the following formula: ESP (%) = (100 × ENa^+^)/CEC.

Determination of eight major ions in soil: First, 50 g of air-dried soil was added to 250 mm CO_2_ free distilled water according to a soil-water ratio of 5:1, oscillated for 5 min, filtered with a vacuum filter, and the filtrate was collected for ion determination. Na^+^ and K^+^ were determined by flame photometry, Ca^2+^ and Mg^2+^ were determined by ethylenediaminetetraacetic acid (EDTA) titration, and Cl^−^ was determined by silver nitrate titration.

Electrical Conductivity (EC): Soil electrical conductivity was measured using a conductivity meter. Air-dried soil samples (passed through a 2 mm sieve) were weighed (10.00 g) into a 50 mL centrifuge tube. CO_2_-free distilled water was added at a soil-to-water ratio of 1:5 (*w*/*v*). The tubes were capped tightly and shaken horizontally for 5 min to ensure complete mixing. The suspension was then allowed to stand for 30 min and subsequently filtered through a quantitative filter paper. The filtrate was collected, and its electrical conductivity was measured at 25 °C using a calibrated conductivity meter. The results were expressed in microsiemens per centimeter (μS/cm).

Bulk Density: Soil bulk density was determined by the core method using a cutting ring with a known volume (typically 100 cm^3^). The cutting ring was driven vertically into the soil profile at the designated sampling point to ensure it was completely filled with minimal compaction. The ring was carefully excavated, and any excess soil extending beyond the edges of the ring was trimmed off flush with a knife. The mass of the soil core contained within the ring was recorded immediately after collection as the wet weight. The soil core was then transferred to an oven and dried at 105 °C to a constant weight (usually for 24–48 h) to obtain the dry weight. Bulk density was calculated using the following formula and expressed in grams per cubic centimeter (g/cm^3^): Bulk Density (g/cm^3^) = Oven-Dry Weight of Soil (g)/Volume of the Cutting Ring (cm^3^).

Soil porosity : Porosity = (1 − soil bulk density/soil specific weight) × 100%

#### 2.2.4. Soil Total DNA Extraction and High-Throughput Sequencing

Genomic DNA from the soil samples was extracted using the E.Z.N.A. Soil DNA Kit (Omega Bio-tek, Inc., Norcross, GA, USA). DNA quality and concentration were determined using a Nanodrop 2000 (ThermoFisher Scientific, Inc., Waltham, MA, USA). For bacteria, primers 338 (5′-ACTCCTACGGGAGGCAGCAG-3′) and 806R (5′-GGACTACNNGGGTATCTAAT-3′) were used to amplify the V3-V4 region of the bacterial 16S ribosomal RNA gene; for fungi, primers ITS1F (5′-CTTGGTCATTTAGAGGAAGTAA-3′) and ITS2 (5′- TGCGTTCTTCATCGATGC-3′) were used for PCR amplification of the ITS1-ITS2 region. PCR products from the same sample were mixed, detected by 1% agarose gel electrophoresis, and purified using the Agencourt AMPure XP Nucleic Acid Purification Kit. Libraries were constructed and quality-checked, followed by paired-end sequencing using the Illumina MiSeq PE300 high-throughput sequencing platform.

#### 2.2.5. Data Processing and Analysis

The raw data obtained from high-throughput sequencing were split, spliced, quality-controlled, and filtered to remove chimeras, resulting in optimized sequences. Sequences with ≥97% similarity were classified as the same operational taxonomic unit (OTU). R (v3.6.0) software was used for plotting, with Venn diagrams showing the number of shared and unique OTUs among samples and rarefaction curves used to assess the rationality of sample sequencing data. QIIME1 (v1.8.0) software was used to calculate alpha diversity indices (including Chao1, Simpson, and Shannon indices) to study the diversity and richness of soil microbial communities in each sample. RDA analysis diagrams were generated using Canoco5 (v4.5) software for RDA (Redundancy analysis) to explore the effects of different environmental factors on the composition of soil microbial communities. SPSS 27.0 software was used for significance analysis of differences.

## 3. Analysis 

### 3.1. The Synergistic Effect of Biochar and Nitrogen Fertilizer on the Regulation of Rhizosphere Soil Properties of Pepper

#### 3.1.1. Regulation of Biochar and Nitrogen Fertilizer on Soil Physical and Chemical Characteristics

The application of biochar and nitrogen fertilizer significantly changed the physical and chemical environment of pepper rhizosphere soil (Table 2). The soil pH reached the highest value of 6.59 under B1N2 treatment, which was significantly higher than that of all other treatments. The application of biochar had the greatest increase in electrical conductivity (EC), and the values (334.67 and 322.67 μS/m) of B1N2 and B1N3 treatments were the highest, which were 13.06% and 9.01% higher than those of the control, respectively. Soil physical properties were markedly improved with biochar amendment. Porosity was significantly increased under the B1N2 treatment, showing a 31.03% improvement compared to the control. Conversely, bulk density was significantly reduced by biochar application, with the B1N0, B1N1, and B1N1 treatments showing reductions of 20.69%, 13.69%, and 6.05%, respectively, compared with B0N0.

The combined application of biochar and nitrogen fertilizer had a significant impact on the salt content and organic matter of pepper rhizosphere soil. The salt content of B1N3 was the lowest at 2.99 g/kg, which was significantly lower than that of the other treatments, a decrease of 27.73% compared with B0N0. Moreover, the addition of biochar in the B1 treatment significantly reduced the salt content of pepper rhizosphere soil, and the salt content was the lowest when the application rate of nitrogen fertilizer was the N3 treatment. For the organic matter content of pepper rhizosphere soil, the N3 treatment had the highest content at 15.24 g/kg, followed by the N2 treatment at 14.99 g/kg, while the N0 treatment had the lowest porosity at only 14.72 g/kg, which was significantly lower than that of the other treatments. The B1 treatment had a significantly higher content of 15.21 g/kg compared to B0. Among the different treatment combinations, B1N3 had the highest organic matter content at 15.49 g/kg, followed by B1N1 at 15.23 g/kg. There was a significant difference between the two, and both were significantly higher than the other treatments.

#### 3.1.2. Effects of Different Biochar and Nitrogen Fertilizer Combined Application on Soil Nutrient Effectiveness

The combined application of biochar and nitrogen fertilizer significantly enhanced soil nutrient availability (Table 3). The total nitrogen content responded strongly to nitrogen application, with the highest values observed in the N3 treatment. The B0N3 and B1N3 treatments showed the highest total nitrogen content (2.02 and 2.00 g/kg, respectively), which was significantly greater than that of the other treatments. The alkaline hydrolyzable nitrogen content followed a similar pattern, with the B1N3 treatment achieving the highest value (150.68 mg/kg). Biochar amendment significantly increased the alkaline hydrolyzable nitrogen content compared to the corresponding treatments without biochar. The available phosphorus content was significantly influenced by both biochar and nitrogen application. The highest available phosphorus content (14.15 mg/kg) was observed in the B1N3 treatment, representing a 6.58% increase over the control (B0N0). Similarly, the available potassium content reached its maximum in the B1N3 treatment (251.63 mg/kg), which was significantly higher than that in all other treatments and represented an 8.87% increase compared to B0N0. These results demonstrate that the combined application of biochar and nitrogen fertilizer, particularly at higher nitrogen levels, significantly improves soil nutrient availability, with biochar enhancing the retention and availability of key nutrients in the soil.

#### 3.1.3. Effects of Different Biochar and Nitrogen Fertilizer Combined Application on Soluble Salt Ion Content in Pepper Rhizosphere Soil

As shown in Table 4, both nitrogen (N) application and biochar (B) application had extremely significant effects on the content of each ion. nitrogen fertilizer and biochar could increase the contents of K^+^, Ca^2+^ and Mg^2+^, and decrease the contents of Na^+^ and Cl^−^; the combined application of nitrogen fertilizer and biochar had a significant effect on the content of K^+^, among which the B1N3 treatment significantly increased the content of K^+^, which was 55.61% higher than that of the B0N0 treatment; meanwhile, the combined application of nitrogen fertilizer and biochar also had a significant effect on the contents of Ca^2+^ and Mg^2+^, and the B1N3 and B0N3 treatments significantly increased the contents of Ca^2+^ and Mg^2+^, which were 23.61%, 156.82% and 22.92%, 182.95% higher than those of the B0N0 treatment, respectively.

In addition, the combined application of nitrogen fertilizer and biochar had a significant effect on the content of Na^+^, with the B1N3 treatment significantly reducing the content of Na^+^ to the lowest level, which was 55.08% lower than that of the B0N0 treatment, followed by the B1N2 and B0N3 treatments, both of which were 39.35% lower than that of the B0N0. For Cl^−^ content, all treatments had a significant decrease compared with the B0N0 treatment, with the B1N3 treatment being the lowest at 0.78 g/kg, which was significantly lower than other treatments, 50.56% lower than the B0N0 treatment, followed by the B1N2 and B0N3 ones.

### 3.2. Effects of Different Biochar and Nitrogen Fertilizer Combined Applications on Pepper Fruit Yield and Quality

As shown in Table 5, biochar application (B), nitrogen fertilizer level (N), and their interaction (B × N) significantly affected pepper yield and various quality indicators. Pepper yield significantly increased with an increase in the application rate of biochar or nitrogen fertilizer. At the same biochar level, the application of nitrogen fertilizer significantly increased the yield; at the same nitrogen fertilizer level, the yield of the treatment with biochar added (B1) was significantly higher than that of the corresponding treatment without biochar added (B0). Among them, the B1N2 treatment had the highest yield, reaching 24,854.1 kg/hm^2^, followed by the B1N3 treatment (24,341.55 kg/hm^2^). There was no significant difference between the two, but both were significantly higher than the other treatments. Compared with the control (B0N0), the yields of B1N2 and B1N3 increased by 42.35% and 39.42%, respectively. The B1N2 and B1N3 treatments showed the highest yields, with no significant difference between them. However, B1N2 was selected as the optimal treatment due to its lower nitrogen input and comparable performance.

The calculation of nitrogen partial factor productivity (PFPN) revealed significant differences among the treatments. As expected, PFPN decreased with increasing nitrogen application rates under the same biochar level, owing to the law of diminishing returns. However, at each corresponding nitrogen level, the addition of biochar significantly enhanced PFPN. The B1N2 treatment, despite being a medium nitrogen input, achieved a remarkably high PFPN of 66.3 kg/kg, which was not significantly lower than the high-nitrogen B1N3 treatment but was achieved with only 56% of the nitrogen fertilizer input. This demonstrates that the combined application of biochar and nitrogen fertilizer not only increases yield, but also dramatically improves the economic and environmental efficiency of nitrogen fertilizer use. The enhancement in PFPN can be attributed to the ability of biochar to reduce nitrogen loss through leaching or volatilization, improve soil physicochemical properties, and promote root development, thereby facilitating greater nutrient uptake and utilization by pepper plants.

The combined application of biochar and nitrogen fertilizer significantly affected the organic acid content in fruits. The B1N2 treatment had the highest organic acid content of 0.37%, followed by the B1N3 treatment with a content of 0.34%, and there was no significant difference between the two; the B0N0 treatment had the lowest organic acid content of 0.18%, which was significantly lower than those of the B1N2 and B1N3 treatments. The combined application of biochar and nitrogen fertilizer significantly increased the soluble protein and soluble sugar content in pepper fruits. The B1N2 treatment had the highest soluble protein content of 33.98 mg/100 g, followed by B1N3 with 32.87 mg/100 g, and there was no significant difference between the two. B0N0 treatment had the lowest soluble protein content, which was significantly lower than that of the other treatments. The soluble sugar content varied significantly among the different treatments. The B1N2 treatment had the highest soluble sugar content of 2.35%, followed by B1N3 with 2.24%, and there was no significant difference between the two. B0N0 treatment resulted in the lowest soluble sugar content. Compared with B0N0, the soluble sugar contents in B1N2 and B1N3 treatments increased by 51.18% and 44.11%, respectively.

Additionally, the combined application of nitrogen fertilizer and biochar significantly affected the vitamin C content in pepper fruits. The B1N2 treatment had the highest vitamin C content of 52.74 mg/100 g, followed by the B1N3 treatment with 51.39 mg/100 g, and there was no significant difference between the two treatments. The B0N0 treatment had the lowest content, which was significantly lower than that of the other treatments. The B1N2 and B1N3 treatments increased by 39.16% and 35.61%, respectively, compared with B0N0. Both biochar and nitrogen fertilizer significantly increased the free amino acid content in pepper fruits, with the application of biochar having a significant effect. B1N3 treatment had the highest content of 685.39 μg/100 g, which was significantly higher than that of the other treatments, and increased by 9.53% compared with the control B0N0 treatment. In conclusion, the application of biochar or nitrogen fertilizer can significantly increase pepper yield and improve pepper fruit quality.

### 3.3. OTU Cluster Analysis of Pepper Rhizosphere Soil Under Combined Application of Different Biochar and Nitrogen Fertilizer

**Figure 1:**
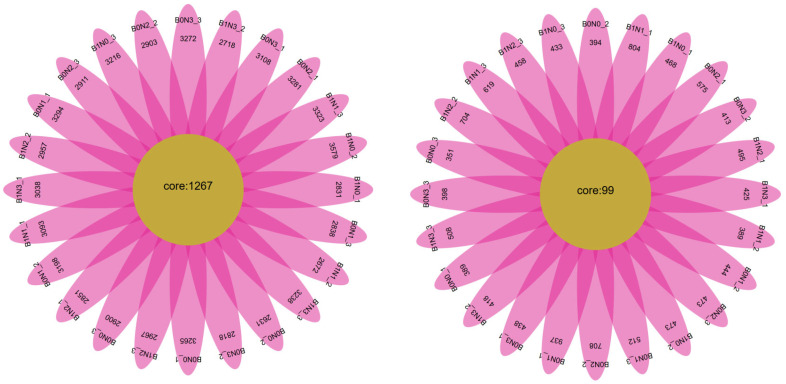
Flowe diagram of OTUs of bacteria (**left**) and fungi (**right**).

**Figure A1:**
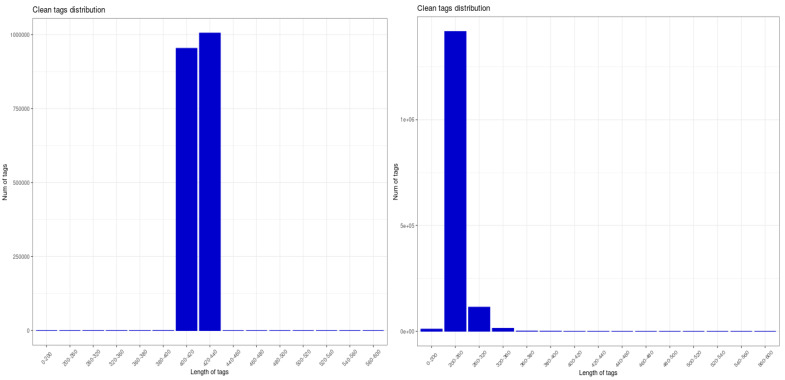
Distribution map of high-quality sample sequences of bacteria (**left**) and fungi (**right**).

As shown in Figure A1, based on the statistical analysis of sequences from high-throughput sequencing results, 1,005,584 valid sequences were obtained from the bacterial 16S rRNA sequencing of soil samples, with lengths ranging from 400 to 440 bp. After OTU clustering at a 97% similarity level, 11,247 bacterial OTUs were identified. For fungal ITS rDNA sequencing, 1,416,791 valid sequences were obtained, with lengths mainly ranging from 0 to 360 bp, and 3200 fungal OTUs were obtained through cluster analysis at a 97% similarity level. As shown in Figure 1, the OTU numbers of bacteria in 24 rhizosphere soil samples (three replicates for each of the eight treatments) are presented. Among the 24 rhizosphere soil samples, the number of shared bacterial OTUs was 1267, accounting for 1.80% of the total, and the number of shared fungal OTUs was 99, accounting for 0.81%.

### 3.4. Analysis of the Effects of Different Biochar and Nitrogen Fertilizer Combined Applications on Microbial Diversity in Pepper Rhizosphere Soil

As shown in Table 6, the application of biochar (B) significantly increased the number and richness of bacterial and fungal species. Nitrogen application (N) significantly affected all fungal diversity indices (observed species, Chao1, Simpson, and Shannon), but only affected the richness index (Chao1) for bacteria. In terms of soil bacteria, among the eight pepper rhizosphere soil samples, the number of detected species and bacterial community richness (Chao1) were the highest in the B1N2 rhizosphere soil. For bacterial communities, the B1N2 treatment showed the highest species richness (Chao1 index), representing a 36.9% increase compared to the control (B0N0). The observed species count followed a similar pattern, with B1N2 showing the highest value. While the Shannon and Simpson indices showed no significant differences among the treatments for bacteria, indicating similar diversity levels, the enrichment in species richness was particularly notable in biochar-amended soils.

In terms of soil fungi, fungal communities responded more strongly to the treatments. The B1N2 treatment again showed the highest values for observed species, Chao1 richness, and Simpson index, representing increases of 50.52%, 30.56%, and 19.84%, respectively, compared to B0N0. The Shannon index was highest in B0N1, but not significantly different from those of the B0N2 and B1N2 treatments. These results demonstrate that the combined application of biochar and nitrogen fertilizer, particularly at the B1N2 level, effectively enhances microbial community richness in the pepper rhizosphere, with more pronounced effects on the fungal communities. The porous structure of biochar provides microbial habitats, thereby increasing the number of species, and can adsorb nutrients for slow release to maintain community stability. An appropriate amount of nitrogen (N2) can promote microbial growth, but excessive nitrogen (N3) can change the soil pH or osmotic pressure, thereby inhibiting some bacterial groups. When nitrogen fertilizer is applied in combination with biochar, biochar fixes nitrogen, reduces leaching, prolongs the validity period of nitrogen nutrients, and enables more sufficient community development.

### 3.5. Analysis of Microbial Community Composition in Pepper Rhizosphere Soil Under Combined Application of Different Biochar and Nitrogen Fertilizer

High-throughput sequencing of the 16S rRNA gene and ITS region provided detailed insights into the structural composition of bacterial and fungal communities inhabiting the pepper rhizosphere under different fertilization regimes.

**Figure 2:**
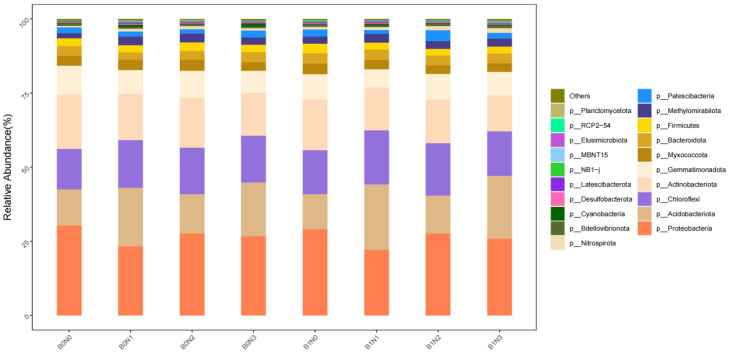
Relative abundance of bacterial communities at the phylum level.

Bacterial Community Structure: As shown in Figure 2, at the phylum level, the bacterial consortia were dominated by *Proteobacteria* (22.14–30.33%), *Acidobacteriota* (11.77–22.12%), *Chloroflexi* (13.55–18.23%), and *Actinobacteriota* (12.13–18.45%), which collectively accounted for a substantial proportion of relative abundance. Other notable phyla included *Gemmatimonadota*, *Myxococcota*, *Bacteroidetes*, *Firmicutes*, *Methylomir*. The application of biochar and nitrogen fertilizer significantly altered the relative abundance of these key phyla. For instance, treatments incorporating biochar (B1), particularly B1N1 and B1N2, led to a pronounced enrichment of *Chloroflexi* and *Bacteroidota* compared with the non-biochar control (B0N0). In contrast, the highest abundances of *Proteobacteria* and *Actinobacteriota* were observed in the B0N0 treatment, suggesting that the addition of amendments modulated the community structure by reducing the dominance of these taxa in favor of other functional groups.

**Figure 3:**
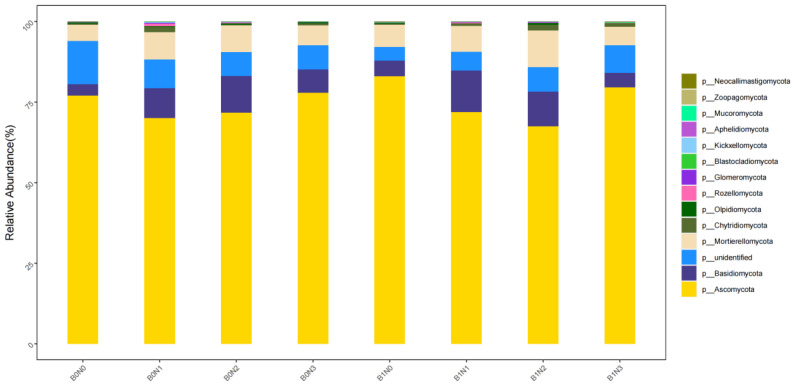
Relative abundance of fungi communities at the phylum level.

Fungal Community Structure: As shown in Figure 3, the fungal community predominantly comprised *Ascomycota* (67.48–83.01%), followed by *Basidiomycota* (3.48–12.89%), *unidentified* (4.28–13.46%), *Mortierellomycota* (4.99–11.43%), and *Chytridiomycota* (0.20–1.72%). Combined amendments induced a notable shift in fungal composition. B1N2 treatment, which yielded the best agronomic performance, was associated with a relative decrease in the abundance of *Ascomycota* and a significant increase in the abundance of *Mortierellomycota* and *Basidiomycota*. This shift suggests a potential functional transition within the fungal community, possibly towards a greater role in nutrient mobilization and soil organic matter decomposition, which may underpin the enhanced soil fertility and plant growth observed in this treatment.

These findings illustrate that the structure of both bacterial and fungal communities in the pepper rhizosphere is highly sensitive to the combined application of biochar and nitrogen fertilizer. The specific enrichment of certain phyla known for their roles in nutrient cycling (e.g., Chloroflexi for carbon metabolism and Mortierellomycota for phosphate solubilization) indicates a functional restructuring of the microbiome, which likely contributes to the observed improvements in soil health and plant productivity.

### 3.6. Correlation Analysis Between Microbial Community Composition and Soil Physicochemical Properties

**Figure 4:**
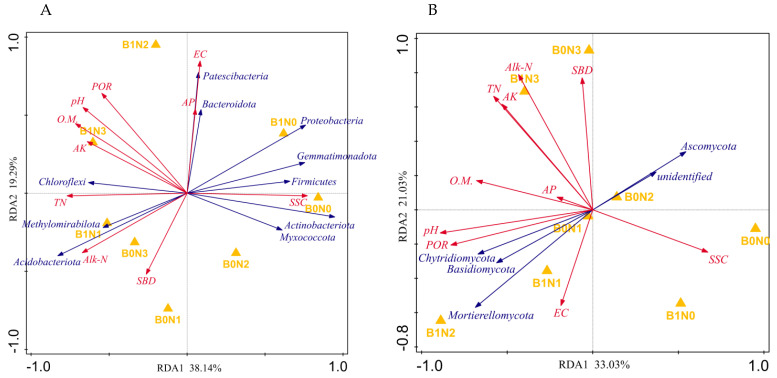
RDA among microbial community structure at the phylum level and soil physico-chemical properties ((**A**) Bacteria; (**B**) Fungi)). Note: Dots represent matrix samples; red arrows represent matrix physicochemical properties; blue arrows represent matrix microorganisms. When the angle between the influencing factors (between the factors and samples) is acute, it indicates a positive correlation between the two factors, and when it is obtuse, it indicates a negative correlation. The longer the ray, the greater is the effect of the factor. pH: pH; EC: electrical conductivity; o.m.: organic matter; TN: total nitrogen; AP: available phosphorus; AK: Fast-acting potassium; Alk-N: alkaline hydrolyzable nitrogen; SSC: salt content; SBD: soil bulk density; POR: porosity.

As shown in Figure 4, the first (38.14%) and second (19.29%) ordination axes cumulatively explained 57.43% of the variation in soil bacterial structure, and the first (33.03%) and second (21.03%) ordination axes of the RDA analysis of fungi and soil physicochemical properties together explained 54.06% of the species variation, indicating that soil physicochemical properties can explain the impact on soil microbial community structure. Among them, pH, EC, O.M., and POR were the dominant factors leading to changes in the bacterial community at the phylum level, while Alk-N, pH, and TN were the dominant factors leading to changes in the fungal community at the phylum level.

In the bacterial community, pH, O.M., and POR were positively correlated with *Chloroflexi*, *Patescibacteria*, *Bacteroidota*, *Methylomirabilota*, and *Acidobacteriota*, and negatively correlated with *Proteobacteria*, *Actinobacteriota*, *Firmicutes*, *Gemmatimonadota*, and *Myxococcota*. EC was positively correlated with *Patescibacteria*, *Bacteroidota*, *Proteobacteria*, *Gemmatimonadota*, and *Firmicutes* and negatively correlated with *Actinobacteria*, *Myxococcota*, *Chloroflexi*, *Methylomirabilota*. In the fungal community, Alk-N was positively correlated with *Ascomycota* and *unidentified*, and negatively correlated with *Chytridiomycota*, *Mortierellomycota*, and *Basidiomycota*; pH and TN were positively correlated with *Chytridiomycota*, *Mortierellomycota*, and *Basidiomycota*, and negatively correlated with *Ascomycota* and *unidentified*.

## 4. Discussion

The application of biochar in agriculture offers a promising strategy for enhancing soil health and crop productivity while mitigating environmental impacts [29]. Corn stover-derived biochar, applied at 0.7% soil mass, combined with 375 kg/hm^2^, showed the most promising results in improving pepper yield and quality. Corn straw biochar promotes growth and improves crop productivity by increasing soil porosity, reducing bulk density, improving soil texture and tillage properties, and enhancing root exudate secretion and rhizosphere microbial activity [33,34,35,36]. All treatments received a balanced basal application of phosphorus and potassium to ensure non-limiting conditions for these nutrients. This design allowed us to isolate the effects of biochar and nitrogen fertilizer interactions without confounding variations in P and K availability. The results of this study showed that the treatment with 0.7% biochar combined with 375 kg/hm^2^ pure nitrogen (B1N2) had the optimal effect, significantly increasing the yield, soluble sugar, soluble protein, vitamin C, free amino acid, and organic acid contents of pepper, and had a significant regulatory effect on the growth and development of pepper. Compared with the treatment without biochar addition, biochar application effectively increased various quality indicators and total yield, which was related to biochar improving the soil environment, promoting nutrient absorption and utilization, and possibly affecting the secondary metabolic pathway of pepper. This result was consistent with the studies by Wu [30], Duan [37], Osama [38], and Chen [39] on improving pepper quality and yield using different types of biochar or combined application with nitrogen fertilizer.

Hossain et al. pointed out that the effect of biochar on crops mainly stemmed from its effective improvement of soil, and its inherent characteristics (such as changing physical and chemical properties and retaining water and fertilizers) played a key role [40]. The addition of biochar can increase soil EC and soil organic carbon (SOC), improve soil quality and physical structure, and be coupled with inorganic fertilizers to enhance nutrient effectiveness, thereby promoting crop growth and yield [28,41]. Second, biochar is rich in mineral nutrients such as phosphorus, potassium, calcium, magnesium, and nitrogen, and can restore soil fertility and enhance productivity and nutrient levels through various mechanisms, the effects of which are mainly mediated by its own characteristics and indirect effects on soil physical and chemical properties and microorganisms [42]. In addition, the positive and negative charges on the surface of biochar can effectively adsorb soil nutrients and reduce the leaching loss of nutrients, such as alkali-hydrolyzable nitrogen, available phosphorus, and Fast-acting potassium [43]. Chen et al. showed that increasing the amount of biochar in sandy loam could gradually improve soil structure (increase porosity and decrease bulk density) and significantly increase water holding capacity [44]. Biochar directly reduces soil bulk density owing to its high porosity and large specific surface area [45]. Its strong adsorption capacity can increase humus content through complexation reactions, promote the formation of soil aggregates, improve aeration and permeability, and efficiently adsorb and fix salt ions on its surface or in pores, thereby reducing the salt concentration in the soil solution [46,47,48].

In this study, it was observed that compared with the treatment without biochar addition, the application of biochar significantly reduced soil bulk density and salt content, increased porosity, organic matter content, and EC value, enhanced soil microbial activity in the root zone, and significantly increased the contents of soil nutrients such as organic matter, alkali-hydrolyzable nitrogen, available phosphorus, and Fast-acting potassium. This indicates that biochar improves soil nutrient supply capacity and root-layer nutrient retention efficiency. The mineral nutrients it contains and its promoting effect on phosphorus effectiveness together improve soil fertility and ultimately significantly increase pepper yield, soil use efficiency, and fruit quality, which is consistent with previous research results. Notably, the biochar treatment had no significant effect on soil pH in this study, which is consistent with the findings of Wang [49], Mao [50], and Zhang [51]. Compared with the control (B0N0), the contents of K^+^, Ca^2+^, and Mg^2+^ increased to varying degrees under different treatments, while the contents of Na^+^ and Cl^−^ in the biochar-added treatment significantly decreased. This result is consistent with other studies, indicating that the surface of biochar is rich in functional groups such as carboxyl and hydroxyl groups, which can adsorb Na^+^ and Cl^−^ through ion exchange, and simultaneously release multivalent cations such as K^+^, Ca^2+^ and Mg^2+^, and its porous structure is also conducive to salt leaching [52]. The improvement in soil physicochemical properties under the B1N2 treatment provides mechanistic explanations for the observed plant growth responses. The increased porosity (by 31.03% compared to B0N0) and reduced bulk density created a more favorable root growth environment, facilitating better root penetration and oxygen supply. The enhanced organic matter content and nutrient availability (particularly alkaline hydrolyzable nitrogen and available phosphorus) created a nutrient-rich rhizosphere environment that supported pepper plant growth and development. These physicochemical improvements collectively contributed to the enhanced nutrient uptake and utilization efficiency observed in our study.

The porous structure of biochar can provide habitats for microorganisms, and its surface functional groups can adsorb nutrient cations, improve soil cation exchange capacity, and fix nutrients for microbial growth [53,54]. As a key driver of soil quality, the structure and function of microbial communities often change significantly due to soil matrix alterations caused by biochar amendment [55]. Many studies have shown that the addition of biochar to soil can improve microbial production efficiency, optimize population structure, and promote rhizosphere microbial growth by improving pH, aeration, and water retention [56,57,58]. Some studies have suggested that the addition of biochar significantly increases the overall richness and diversity of soil microbial communities [59,60]. In addition, the results of this experiment showed that B1N2 treatment significantly increased the richness (observed species, Chao1) of soil bacterial and fungal communities and the dominance (Simpson) of fungal communities. The porous structure of biochar provides additional microbial habitats, and its large specific surface area and surface functional groups can adsorb nutrients and release them slowly, which helps maintain a stable and diverse microbial community [53,54,59]. An appropriate nitrogen supply (N2 level) provides an essential nitrogen source for microbial growth, whereas excessive nitrogen (N3) may inhibit some microorganisms by altering soil pH or causing osmotic stress [56], which explains why the microbial diversity index of the B1N3 treatment was not optimal in this experiment. The increased microbial diversity under B1N2 treatment, particularly for fungi (Chao1 index increased by 30.56% compared to B0N0), can be attributed to the dual effect of biochar and optimal nitrogen supplementation. Biochar provides a porous habitat for microbial colonization while its surface functional groups adsorb and slowly release nutrients, creating a stable and diverse microbial environment. The moderate nitrogen level (N2) provides essential nutrients without causing the osmotic stress or pH changes associated with excessive nitrogen application (N3).

This study identified the dominant bacteria (*Proteobacteria*, *Acidobacteriota*, *Chloroflexi*, *Actinobacteriota*, *Gemmatimonadota*, *Myxococcota*, *Bacteroidota*, *Firmicutes*, *Methylomirabilota*, *Patescibacteria*) and fungal phyla (*Ascomycota*, *Basidiomycota*, *unidentified*, *Mortierellomycota*, *Chytridiomycota*) in pepperrhizosphere soil. Redundancy analysis (RDA) further revealed the key soil factors driving changes in microbial communities: the bacterial community was mainly regulated by pH, organic matter, and porosity, whereas the fungal community mainly responded to changes in alkali—hydrolyzable nitrogen and total nitrogen. These factors together constitute the rhizosphere microenvironment, shaping the community structure and function of microorganisms by affecting their survival resources (nutrients, water, oxygen) and living conditions (pH, osmotic pressure) [55,61], confirming that soil physical and chemical properties are the key factors affecting the distribution of rhizosphere microorganisms. The significant enrichment of *Chloroflexi* and *Mortierellomycota* is particularly noteworthy. *Chloroflexi* are known for their role in carbon cycling and organic matter decomposition, which aligns with the increased soil organic matter content we observed. *Mortierellomycota* species are recognized as efficient phosphate solubilizers, explaining the enhanced phosphorus availability in the B1N2 treatment. These functional shifts in the microbial community directly contribute to the improved soil fertility and plant growth.

**Figure 5:**
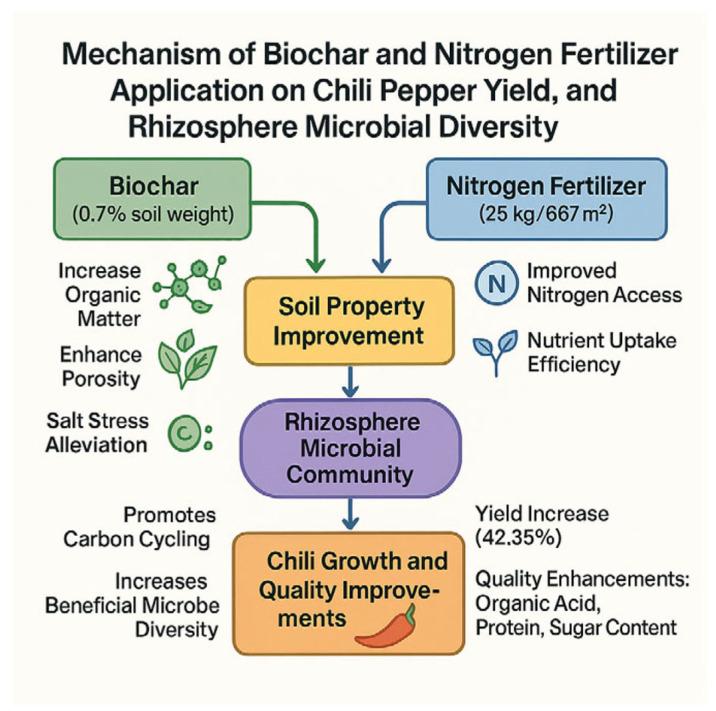
Proposed mechanistic model illustrating the synergistic effects of combined biochar and nitrogen fertilizer application on pepper growth and soil health.

The integration of our findings allows us to propose a cohesive mechanistic model that elucidates how the combined application of biochar and optimal nitrogen fertilizer (B1N2) synergistically enhances pepper productivity by modifying the soil environment and microbial functionality. This model is conceptualized in Figure 5, The combined application of biochar and nitrogen fertilizer synergistically enhances pepper yield and quality through integrated improvements in soil physical structure (increased porosity, reduced bulk density), chemical nutrient retention (reduced N leaching, increased organic matter and CEC), and biological activity (enrichment of functional microbes like Chloroflexi and Mortierellomycota for nutrient cycling). These optimizations collectively promote root development, nutrient uptake, and fruit production, demonstrating that biochar and N fertilizer together create a sustainable soil–plant environment that supports higher productivity and efficiency than either amendment alone.

Although biochar played a key role, the amount of nitrogen fertilizer had a significant effect on the results. At the N2 level, the yield and quality parameters generally increased with the increase of nitrogen application rate, but no significant further improvement was observed after exceeding the N2 level, indicating the optimal range of nitrogen input. It is worth noting that biochar itself contributes about a portion of nitrogen, although this nitrogen may initially be stubborn, but it may contribute to the long-term nitrogen pool and soil fertility, partially explaining the positive effects observed in B1N0 treatment compared to the absolute control (B0N0).

## 5. Results

The results of this study indicate that the combined application of corn stover biochar (0.7% soil mass) and an appropriate amount of nitrogen fertilizer (375 kg/hm^2^ pure nitrogen) (B1N2) is the optimal strategy to increase pepper yield, improve fruit quality, ameliorate the rhizosphere soil environment, and enhance microbial community function. This combination exerts a synergistic effect through multiple pathways: (1) significantly improving soil physical structure (reducing bulk density and increasing porosity), promoting root development and water-fertilizer transport; (2) effectively increasing the contents and retention capacity of soil organic matter and nutrients such as nitrogen, phosphorus, and potassium, ensuring the nutritional requirements of pepper; (3) alleviating salt stress (reducing Na^+^ and Cl^−^ contents and optimizing ion composition); (4) enriching beneficial microbial groups with carbon cycling and phosphorus-solubilizing functions, and increasing the richness of microbial communities, thereby enhancing the efficiency of soil nutrient biotransformation and supply. These effects collectively promote the growth, yield formation, and fruit quality improvement of pepper plants. These findings have significant implications for sustainable agricultural practices. The B1N2 strategy not only increases pepper productivity and quality but also reduces the environmental footprint of nitrogen fertilization by enhancing use efficiency and fostering a healthier soil ecosystem. This approach can be particularly valuable in intensive vegetable production systems aiming to achieve both high output and environmental sustainability.

## Figures and Tables

**Table 1:** The ratio of biochar to pure nitrogen application.

Handling Names	Proportion of Biochar Application (%)	The Ratio of Pure Nitrogen Application (kg/hm^2^)
B0N0 (control)	0	0
B0N1	0	75
B0N2	0	375
B0N3	0	675
B1N0	0.7	0
B1N1	0.7	75
B1N2	0.7	375
B1N3	0.7	675

**Table 2:** Effects of different biochar and nitrogen fertilizer on soil chemical properties.

Treatments	pH	Electrical Conductivity (μS/m)	Porosity of Substrate (%)	Volume Weight of Soil (g/cm^3^)	Salt Content (g/kg)	Organic Carbon (g/kg)
B0N0	6.05 ± 0.002 ^d^	315.03 ± 2.65 ^d^	50.07 ± 0.58 ^f^	1.15 ± 0.01 ^c^	4.15 ± 0.03 ^b^	14.43 ± 0.03 ^g^
B0N1	6.22 ± 0.02 ^c^	296.01 ± 1.16 ^f^	53.27 ± 0.56 ^e^	1.28 ± 0.02 ^b^	3.05 ± 0.02 ^f^	14.65 ± 0.02 ^f^
B0N2	6.07 ± 0.02 ^d^	282.34 ± 1.86 ^g^	59.14 ± 0.71 ^c^	1.21 ± 0.02 ^c^	4.42 ± 0.03 ^a^	14.89 ± 0.02 ^e^
B0N3	6.28 ± 0.02 ^b^	282.34 ± 0.88 ^g^	54.54 ± 0.41 ^e^	1.36 ± 0.05 ^a^	3.77 ± 0.18 ^c^	14.98 ± 0.02 ^d^
B1N0	6.10 ± 0.01 ^d^	329.02 ± 1.73 ^b^	57.04 ± 0.29 ^d^	0.92 ± 0.01 ^f^	4.56 ± 0.03 ^a^	15.03 ± 0.01 ^d^
B1N1	6.31 ± 0.02 ^b^	308.67 ± 1.74 ^e^	60.14 ± 0.38 ^c^	0.99 ± 0.02 ^e^	3.57 ± 0.02 ^d^	15.23 ± 0.02 ^b^
B1N2	6.60 ± 0.02 ^a^	334.67 ± 2.85 ^a^	65.6 ± 0.38 ^a^	1.08 ± 0.02 ^d^	3.35 ± 0.02 ^e^	15.11 ± 0.05 ^c^
B1N3	6.29 ± 0.02 ^b^	322.67 ± 1.21 ^c^	62.47 ± 0.47 ^b^	1.20 ± 0.01 ^c^	2.99 ± 0.02 ^f^	15.49 ± 0.01 ^a^
ANOVA						
B	**	**	**	**	**	**
N	**	**	**	**	**	**
B × N	**	**	ns	*	**	**

Note: Data are presented as mean ± standard deviation. In the same column, values followed by different lowercase letters indicate significant differences, while the same lowercase letters indicate no significant difference (*p* < 0.05). B, biochar modifier (0 and 0.7% by soil weight); N, nitrogen treatment (pure nitrogen at 0, 75, 375, and 675 kg/hm^2^). ns, no significant difference; * *p* < 0.05; ** *p* < 0.01.

**Table 3:** Effects of different biochar and nitrogen fertilizer on soil nutrient availability.

Treatments	Total Nitrogen (g/kg)	Alkaline Hydrolyzable Nitrogen (mg/kg)	Available Phosphorus (mg/kg)	Fast-Acting Potassium (mg/kg)
B0N0	1.95 ± 0.01 ^e^	137.06 ± 0.35 ^f^	231.14 ± 0.59 ^c^	13.27 ± 0.02 ^d^
B0N1	1.98 ± 0.01 ^bc^	145.72 ± 0.32 ^c^	220.70 ± 0.40 ^d^	13.07 ± 0.03 ^e^
B0N2	1.96 ± 0.01 ^de^	148.81 ± 0.30 ^b^	231.17 ± 0.62 ^c^	13.57 ± 0.02 ^c^
B0N3	2.02 ± 0.01 ^a^	149.97 ± 0.61 ^ab^	210.70 ± 0.38 ^e^	14.06 ± 0.02 ^a^
B1N0	1.96 ± 0.01 ^de^	137.6 ± 0.28 ^f^	221.67 ± 0.91 ^d^	13.34 ± 0.04 ^d^
B1N1	1.98 ± 0.01 ^cd^	143.34 ± 0.52 ^d^	201.10 ± 0.59 ^f^	13.61 ± 0.01 ^c^
B1N2	1.99 ± 0.01 ^bc^	141.07 ± 0.46 ^e^	241.44 ± 0.84 ^b^	13.73 ± 0.01 ^b^
B1N3	2.00 ± 0.01 ^bc^	150.67 ± 0.35 ^a^	251.64 ± 0.89 ^a^	14.15 ± 0.04 ^a^
ANOVA				
B	ns	**	**	**
N	**	**	**	**
B × N	**	**	ns	*

Note: Data are presented as mean ± standard deviation. In the same column, values followed by different lowercase letters indicate significant differences, while the same lowercase letters indicate no significant difference (*p* < 0.05). B, biochar modifier (0 and 0.7% by soil weight); N, nitrogen treatment (pure nitrogen at 0, 75, 375, and 675 kg/hm^2^). ns, no significant difference; * *p* < 0.05; ** *p* < 0.01.

**Table 4:** Effects of combined application of different biochar and nitrogen fertilizer on soluble salt ion content in rhizosphere soil of pepper.

Treatments	K^+^ (g/kg)	Na^+^ (g/kg)	Ca^2+^ (g/kg)	Mg^2+^ (g/kg)	Cl^−^ (g/kg)
B0N0	4.26 ± 0.12 ^g^	0.75 ± 0.01 ^a^	0.58 ± 0.01 ^e^	0.35 ± 0.01 ^g^	1.60 ± 0.07 ^a^
B0N1	4.54 ± 0.08 ^f^	0.69 ± 0.02 ^b^	0.67 ± 0.01 ^c^	0.72 ± 0.02 ^e^	1.38 ± 0.04 ^b^
B0N2	4.89 ± 0.08 ^e^	0.58 ± 0.02 ^d^	0.68 ± 0.03 ^b^	0.79 ± 0.01 ^d^	1.21 ± 0.18 ^d^
B0N3	5.67 ± 0.12 ^c^	0.46 ± 0.01 ^f^	0.71 ± 0.02 ^a^	0.99 ± 0.03 ^a^	0.96 ± 0.14 ^f^
B1N0	4.89 ± 0.12 ^e^	0.63 ± 0.03 ^c^	0.55 ± 0.01 ^f^	0.34 ± 0.01 ^f^	1.31 ± 0.07 ^c^
B1N1	5.32 ± 0.16 ^d^	0.52 ± 0.03 ^e^	0.62 ± 0.02 ^d^	0.45 ± 0.01 ^d^	1.13 ± 0.04 ^e^
B1N2	6.18 ± 0.12 ^b^	0.45 ± 0.02 ^f^	0.67 ± 0.01 ^c^	0.86 ± 0.02 ^c^	0.96 ± 0.04 ^f^
B1N3	6.65 ± 0.08 ^a^	0.34 ± 0.01 ^g^	0.72 ± 0.03 ^a^	0.9 ± 0.03 ^a^	0.78 ± 0.14 ^g^
ANOVA					
B	**	**	**	**	**
N	**	**	**	**	**
B × N	**	**	**	**	**

Note: Data are presented as mean ± standard deviation. In the same column, values followed by different lowercase letters indicate significant differences, and the same lowercase letters indicate no significant difference (*p* < 0.05). B, biochar modifier (0 and 0.7% by soil weight); N, nitrogen treatment (pure nitrogen at 0, 75, 375, and 675 kg/hm^2^, respectively). ** *p* < 0.01.

**Table 5:** Effects of different biochar and nitrogen fertilizer on yield and quality of pepper fruit.

Treatments	Yield (kg/hm^2^)	PFPN (kg/kg)	Titratable Acid (%)	Soluble Protein (mg/100 g)	Soluble Sugar (%)	Vitamin C Content (mg/100 g)	Free Amino Acid Content (μg/100 g)
B0N0	17,459.7 ± 535.35 ^e^	N/A	0.18 ± 0.01 ^d^	14.26 ± 0.24 ^f^	1.56 ± 0.04 ^d^	37.90 ± 0.71 ^e^	607.52 ± 3.16 ^d^
B0N1	19,769.7 ± 204.3 ^d^	263.6 ± 2.7 ^c^	0.22 ± 0.01 ^c^	16.75 ± 0.25 ^e^	1.79 ± 0.04 ^c^	40.35 ± 0.40 ^d^	627.75 ± 3.34 ^cd^
B0N2	21,850.5 ± 258.45 ^c^	58.3 ± 0.7 ^d^	0.24 ± 0.01 ^c^	21.72 ± 0.65 ^d^	2.05 ± 0.05 ^b^	47.63 ± 0.45 ^bc^	634.92 ± 2.12 ^c^
B0N3	22,993.2 ± 262.65 ^c^	34.1 ± 0.4 ^e^	0.29 ± 0.02 ^b^	27.52 ± 0.40 ^b^	2.01 ± 0.05 ^b^	47.96 ± 0.82 ^b^	649.09 ± 4.85 ^b^
B1N0	19,546.35 ± 442.35 ^d^	N/A	0.21 ± 0.02 ^cd^	17.02 ± 0.80 ^e^	1.61 ± 0.01 ^d^	39.83 ± 0.12 ^d^	616.58 ± 1.20 ^de^
B1N1	22,572.45 ± 193.8 ^bc^	301.0 ± 2.6 ^b^	0.27 ± 0.02 ^b^	24.88 ± 0.58 ^c^	1.83 ± 0.05 ^c^	46.14 ± 0.85 ^c^	628.91 ± 2.09 ^cd^
B1N2	24,854.1 ± 458.4 ^a^	66.3 ± 1.2 ^c^	0.37 ± 0.02 ^a^	33.98 ± 0.74 ^a^	2.35 ± 0.09 ^a^	52.74 ± 0.77 ^a^	655.17 ± 5.53 ^b^
B1N3	24,341.55 ± 335.85 ^a^	36.1 ± 0.5 ^e^	0.34 ± 0.04 ^a^	32.87 ± 0.90 ^a^	2.24 ± 0.07 ^a^	51.39 ± 0.60 ^a^	685.39 ± 6.58 ^a^
ANOVA							
B	**	**	*	**	**	**	**
N	**	**	**	**	**	**	**
B × N	**	**	**	**	**	**	**

Note: Data are presented as mean ± standard deviation (SD). In the same column, values followed by different lowercase letters indicate significant differences, while the same lowercase letters indicate no significant difference (*p* < 0.05). B, biochar modifier (0 and 0.7% by soil weight); N, nitrogen treatment (pure nitrogen at 0, 75, 375, and 675 kg/hm^2^). * *p* < 0.05, ** *p* < 0.01. N/A: Not Applicable.

**Table 6:** Microbial diversity indexes in the soil samples.

Microbiome	Sample Name	Observed Species Index	Chao1 Index	Simpson Index	Shannon Index
Bacteria	B0N0	3865.61 ± 184.02 ^b^	4039.15 ± 368.03 ^b^	1 ^a^	9.92 ± 0.17 ^a^
	B0N1	4376.8 ± 138.9 ^a^	4557.92 ± 136.49 ^ab^	1 ^a^	10.02 ± 0.05 ^a^
	B0N2	4298.63 ± 124.7 ^ab^	4393.58 ± 221.18 ^ab^	1 ^a^	10.02 ± 0.07 ^a^
	B0N3	4199.52 ± 89.08 ^ab^	4472.32 ± 167.64 ^ab^	1 ^a^	9.99 ± 0.03 ^a^
	B1N0	4308.7 ± 112.65 ^ab^	4264.01 ± 348.85 ^b^	1 ^a^	10.05 ± 0.13 ^a^
	B1N1	4429.69 ± 80.38 ^a^	4919.83 ± 190.77 ^ab^	0.99 ^a^	9.83 ± 0.17 ^a^
	B1N2	4592.02 ± 181.05 ^a^	5529.79 ± 224.89 ^a^	1 ^a^	9.92 ± 0.07 ^a^
	B1N3	4398.23 ± 58.14 ^a^	5116.11 ± 174.67 ^ab^	1 ^a^	9.92 ± 0.13 ^a^
Fungi	B0N0	477.02 ± 13.58 ^b^	575.08 ± 17.42 ^b^	0.79 ± 0.03 ^bc^	4.17 ± 0.15 ^b^
	B0N1	563.33 ± 23.92 ^ab^	658.29 ± 20.42 ^ab^	0.94 ± 0.02 ^a^	5.92 ± 0.48 ^a^
	B0N2	607.67 ± 33.19 ^ab^	766.69 ± 57.33 ^a^	0.94 ± 0.01 ^a^	5.76 ± 0.23 ^a^
	B0N3	515.33 ± 11.67 ^b^	598.64 ± 9.98 ^c^	0.89 ± 0.04 ^ab^	5.02 ± 0.43 ^ab^
	B1N0	556.97 ± 12.62 ^ab^	677.85 ± 14.72 ^ab^	0.79 ± 0.06 ^c^	4.32 ± 0.37 ^b^
	B1N1	593.14 ± 64.49 ^ab^	569.41 ± 25.57 ^b^	0.88 ± 0.02 ^abc^	5.16 ± 0.19 ^ab^
	B1N2	718.09 ± 43.82 ^a^	750.79 ± 99.96 ^a^	0.95 ± 0.01 ^a^	5.83 ± 0.08 ^a^
	B1N3	582.67 ± 33.19 ^ab^	628.06 ± 14.301 ^ab^	0.91 ± 0.02 ^a^	5.16 ± 0.39 ^ab^
	ANOVA				
Bacteria	B	*	*	ns	ns
	N	ns	**	ns	ns
	B × N	*	**	ns	ns
Fungi	B	**	ns	ns	ns
	N	**	**	**	**
	B × N	**	*	**	**

Note: Data are presented as mean ± standard deviation. In the same column, values followed by different lowercase letters indicate significant differences, while the same lowercase letters indicate no significant difference (*p* < 0.05). B, biochar modifier (0 and 0.7% by soil weight); N, nitrogen treatment (pure nitrogen at 0, 75, 375, and 675 kg/hm^2^). ns, no significant difference; * *p* < 0.05; ** *p* < 0.01.

## Data Availability

The datasets generated during and/or analyzed during the current study are available from the corresponding author on reasonable request. The data are not publicly available due to the priority to complete the follow-up study.
